# Dietary strategies for optimizing omega-3 fatty acid intake: a nutrient database-based evaluation in Taiwan

**DOI:** 10.3389/fnut.2025.1661702

**Published:** 2025-09-19

**Authors:** Shih-An Lu, I-Ta Lee, Chui Xuan Tan, Shang-Ta Wang, Keerthi Praveen, Wei-Ju Lee

**Affiliations:** ^1^School of Food Safety, College of Nutrition, Taipei Medical University, Taipei, Taiwan; ^2^School of Dentistry, College of Oral Medicine, Taipei Medical University, Taipei, Taiwan; ^3^Department of Food Science, National Taiwan Ocean University, Keelung, Taiwan; ^4^Institute of Food Safety and Risk Management, National Taiwan Ocean University, Keelung, Taiwan; ^5^Department of Chemistry, CEG Campus, Anna University, Chennai, India; ^6^Nutrition Research Center, Taipei Medical University Hospital, Taipei, Taiwan

**Keywords:** Food Nutrient Database, alpha-linolenic acid, eicosapentaenoic acid, docosahexaenoic acid, Food Consumption Database, daily intake

## Abstract

**Introduction:**

Omega-3 fatty acid consumption is increasingly important for overall health.

**Methods:**

This study assessed the effectiveness of different omega-3 sources, including oils, nuts, seeds, and aquatic foods, in a particular cultural setting using information from the Taiwan Food and Drug Administration Food Nutrient Database.

**Results:**

Among edible oils, 22.7% contained omega-3 levels exceeding 0.2 g/g, with flaxseed oil requiring only 2–3 g/day to meet α-linolenic acid (ALA) recommendations of the National Institutes of Health (NIH). Flaxseed, rapeseed, walnut, canola, and soybean oils can meet ALA intake recommendations, with flaxseed and chia seeds being the most efficient sources, requiring only 5–7 g/day. Mackerel and Pacific saury were the most efficient eicosapentaenoic acid (EPA) and docosahexaenoic acid (DHA) sources, requiring only 6 g/day, whereas 81% of crustaceans and 73.3% of mollusks contained <0.01 g/g of omega-3, necessitating much higher intake. Integration with the Food Consumption Database showed that while the 95th percentile and mean ALA intakes exceeded NIH recommendations, median levels fell below, indicating a deficiency risk for over half the population. EPA and DHA intake were generally inadequate, particularly among girls aged 16–18 and children under 3, except in adults and the elderly.

**Discussion:**

These findings underscore the need to promote public awareness of potential omega-3 deficiency.

## 1 Introduction

The role of dietary fats in human health has been a focal point of nutritional research and public health discussions for decades. The main component of edible oil is triacylglycerols, whose chemical structure can be classified into saturated fatty acids (FAs; SFAs) with no double bonds, monounsaturated FAs (MUFAs) with one double bond, and polyunsaturated FAs (PUFAs) with two or more double bonds. The latest updated “Dietary Guidelines” of the World Health Organization (WHO) no longer recommend a specific amount of fat intake but continue to stress limiting saturated fat consumption to < 10% of total daily calories (1). Contemporary evidence underscores the importance of distinguishing between types of dietary fats, particularly emphasizing the health benefits of MUFAs and PUFAs. MUFAs are represented by oleic acid, which is an omega-9 FA, while PUFAs include omega-3 and omega-6 FAs. Among these FAs, linoleic acid (omega-6) and α-linolenic acid (ALA) (omega-3) are recognized as essential FAs for humans. A lower omega-6 to omega-3 FA ratio is more favorable for reducing the risk of many prevalent chronic diseases and obesity (2, 3).

In addition to ALA, eicosapentaenoic acid (EPA), and docosahexaenoic acid (DHA) are also omega-3 FAs. Numerous studies demonstrated that omega-3 FAs, such as EPA and DHA, possess cardiovascular and neurological benefits (4, 5) and prevent preterm and early preterm births (6, 7). The diet and reinfarction trial (DART) trial revealed that consuming oily fish reduced cardiovascular-related mortality by 29%, providing the first clinical evidence that omega-3 FAs can effectively reduce the risk of death in patients with coronary heart disease (8). The GISSI trial further supported those findings, by showing that patients consuming 1 g daily of EPA and DHA experienced a significant reduction in total mortality and sudden cardiac death, with benefits observed at as early as 3 months into treatment. The study demonstrated a 41% relative risk (RR) reduction in total mortality at 3 months and a 53% reduction in sudden death at 4 months. Those results suggest an antiarrhythmic effect of n-3 PUFAs, that contribute to improved survival rates in post-myocardial infarction patients (9). Similarly, the Japan EPA Lipid Intervention Study (JELIS) trial conducted in Japan demonstrated that high dose EPA supplementation (1,800 mg/day) reduced the risk of major coronary artery events by 19%, underscoring the independent protective role of EPA for preventing major coronary events, and especially non-fatal coronary events, in Japanese hypercholesterolemic patients (10). Based on a review by Petsini et al. who collected and compared fish intervention studies, with EPA and DHA intake of 0.03–5 g/day, biomarkers, such as triglycerides, high-density lipoprotein, and platelet aggregation, tended to be ameliorated when daily intake exceeded 1 g/day (11). Collectively, those studies established omega-3 FAs as potent contributors to cardiovascular health, neurological function, and pregnancy outcomes. Compelling evidence from clinical trials underscores the significance of EPA and DHA supplementation in reducing cardiovascular related mortality, preventing arrhythmic events, and lowering the incidence of major coronary events. The dose-dependent effects observed in various studies highlight that a daily intake exceeding 1 g of EPA and DHA may be particularly beneficial in improving lipid profiles and reducing platelet aggregation.

According to the National Institutes of Health (NIH) (12), the recommended daily intake of ALA varies by age group. For infants, it is 0.32 g/day for those aged 0–6 months and 0.5 g/day for those aged 7–12 months. For children, the recommended intake is 0.7 g/day for those aged 1–3 years and 0.9 g/day for those aged 4–8 years. Boys aged 9–13 years should consume 1.2 g/day, while girls in the same age group require 1.0 g/day. For older children and adolescents, boys aged 14–18 years need 1.6 g/day, and girls in the same age group need 1.1 g/day. Adult and elderly men should aim for 1.6 g/day, and adult and elderly women need 1.1 g/day. Pregnant women require 1.4 g/day, and lactating women should consume 1.3 g/day. On the other hand, recommended intake levels of EPA and DHA vary across health organizations (13). Unlike ALA, dietary reference intake (DRI) levels have not been established for EPA and DHA. The WHO advises a combined daily intake of 250–500 mg of EPA and DHA for general health, particularly for cardiovascular health (13). The American Heart Association (AHA) recommends a similar intake of 250–500 mg/day for healthy adults, with higher doses (≥1,000 mg) suggested for individuals with cardiovascular diseases under medical supervision (12). The European Food Safety Authority (ESFA) also recommends a daily intake of 250 mg of combined EPA and DHA to support normal heart function (14). In addition, the 2020–2025 Dietary Guidelines for Americans (DGA) advise that the general population to consume about 8 ounces (~227 g) of a variety of seafood per week, which corresponds to an average daily intake of ~250 mg of EPA and DHA (15). Despite these guidelines, it was estimated that majority of US childbearing-age and pregnant women consumed significantly lower amounts of seafood than what the DGA recommends, which can subsequently lead to low intake levels of EPA and DHA (16).

ALA can be metabolized to EPA and DHA by desaturases and elongases in humans. However, the conversion is limited, making direct dietary sources of EPA and DHA crucial for meeting physiological requirements (17). Previous studies highlighted high ALA contents in foods such as flaxseed oil and the rich EPA and DHA concentrations in oily fish, such as mackerel and salmon (2, 18). In Taiwan, detailed analyses of omega-3 contents across a wide variety of consumer foods were documented in the Food Nutrient Database, encompassing plant-based oils, nuts, and seeds, as well as aquatic foods. While these foods collectively provide a diverse array of omega-3 FAs, their specific contributions to daily intake have not been thoroughly quantified.

The aim of this study was to conduct a comprehensive analysis of omega-3 FA profiles in oils, nuts, seeds, and aquatic foods, utilizing data from the Taiwan Food and Drug Administration (TFDA) Food Nutrient Database (19). Given that this database offers a consistent reference for omega-3 contents across various food categories, the study intended to provide an evidence-based foundation for translating these findings into actionable dietary recommendations. By combining omega-3 contents of major food categories with dietary frequency data from the Taiwan Food Consumption Database, the daily omega-3 intake of different populations can be estimated. This study represents the first attempt to evaluate daily omega-3 nutrient intake using both a nutrition database and a Food Consumption Database. The findings can serve as a basis for calculating the intake of essential nutrients such as omega-3 and provide dietary recommendations based on results of this research.

## 2 Materials and methods

### 2.1 Data collection

This study utilized data from the TFDA Food Nutrient Database (19). A targeted selection process identified foods in Taiwan rich in omega-3 FAs, focusing on oils (*n* = 22), nuts (*n* = 10), seeds (*n* = 8), fish (*n* = 121), crustaceans (*n* = 21), and mollusks (*n* = 15). Nutritional profiles were extracted for each food item, including the total fat content and the composition of saturated FAs (SFAs), MUFAs, and PUFAs, specifically ALA, EPA, and DHA.

The data used in this study were obtained from the TFDA Food Nutrition Database, which primarily provides duplicated data, with limited comparisons across different varieties, processing methods, or cooking techniques (19). Given that the impacts of cooking on fats remain unclear, this study utilized only one set of repeated data for each food category. In cases where both raw and cooked food data were available, only data for raw foods were used. For nuts, the pretreatment varied to reflect common consumption practices in Taiwan. Raw samples were used for walnuts, chia seed, awkeotsang (*Ficus pumila*), and lotus nuts, which were homogenized after preparation. Other nuts and seeds were pretreated as roasted samples before being homogenized.

Sample pretreatments varied depending on the type of seafood being analyzed. For fish species, raw samples were descaled, and their heads, tails, viscera, and bones were removed prior to homogenization into a uniform mixture. Shrimp and lobster samples were prepared by removing the shell, head, tail, and digestive tract before homogenization. In the case of crabs, the shells were removed, and edible parts such as crab legs and meat were isolated for homogenization. Shellfish were prepared by removing the shells, followed by homogenization of the edible parts. For mollusks such as squid, the preparation involved removing the skin and internal cartilage before homogenization.

### 2.2 Calculation of daily minimally required consumption

The omega-3 FA content of each food item was calculated by dividing the total omega-3 content (ALA + EPA + DHA) by the sum of total FAs (SFAs + MUFAs + PUFAs) and multiplying by the total fat content, expressed as a percentage. To determine the quantity of each food item required to meet daily omega-3 intake guidelines, the following parameters were used: daily EPA and DHA requirements of 250, 500, and 1,000 mg/day as recommended by international dietary guidelines, and daily ALA requirements of 1.6 g/day for men and 1.1 g/day for women in reference to the NIH (12, 13). The daily minimally required consumption (g/day) was calculated by dividing the recommended intake levels by the total ALA or EPA and DHA contents in the food to obtain the minimally required amount of food to meet omega-3 requirements. The daily minimally required consumption (g/day) for each food item was then ranked and categorized to enable direct comparisons among different food sources. This ranking highlighted how efficient a food source is to meet health guidelines.

### 2.3 Estimation of the daily intake of omega-3 via the main types of omega-3 foods

Daily dietary intake levels of omega-3 FAs were estimated by integrating food composition data with dietary intake data. ALA is predominantly found in plant-based oils, whereas DHA and EPA are primarily derived from fish and other seafood. Omega-3 concentrations (g/g) in edible oils, nuts, seeds, fish, crustaceans, and mollusks were obtained from the TFDA Food Nutrient Database (19). The mean concentrations of ALA, EPA, and DHA were calculated for each food category.

Dietary intake amounts were derived from the Taiwan Food Consumption Data-base (19), which includes age-stratified records of daily food consumption across categories such as oils and fats, seeds and nuts, and aquatic products. Participants also reported demographic data, including average body weights (BW), stratified into seven age groups: 0 to ≤ 3, >3 to ≤ 6, >6 to ≤ 12, >12 to ≤ 15, >15 to ≤ 19, >19 to ≤ 65, and >65 years. Daily intake of omega-3 FAs from each food category was estimated by multi-plying the amount of food consumed (g/day) by its respective mean omega-3 content (g/g), assuming 100% bioavailability as commonly applied in initial nutrient exposure assessments set by the NIH and WHO.

Two intake scenarios were considered for comparison against dietary reference values provided by the NIH (12) and the WHO (13): (1) which estimated average population intake using mean and median omega-3 concentrations in each food category; and (2) representing a high-intake group, based on the P95 of food consumption data for each category. These intake estimates were then evaluated for adequacy across demographic groups by comparing them to age-specific dietary intake recommendations. This approach enabled population-level estimations of ALA, EPA, and DHA in-take across age groups and dietary habits.

## 3 Results

### 3.1 Edible oils and fats

Table 1 provides a comprehensive ranking of omega-3 FA contents across different oils. In this category, due to the nearly 100% oil contents in oils and fats, the proportion of omega-3 in the oil directly reflects its omega-3 content (Table 1). Flaxseed oil ranked highest with an omega-3 concentration of 0.54 g/g, significantly outperforming other oils. Other oils, such as walnut oil (0.14 g/g) and rapeseed oil (0.10 g/g), also demonstrated notable omega-3 levels, making them valuable dietary choices for meeting ALA intake recommendations. Soybean oil (0.07 g/g) and corn oil (0.03 g/g) also contributed moderate levels of omega-3 FAs, followed by rice bran oil and white sesame oil. It was observed that ALA is also present in animal oils, including chicken oil and lard (Table 1). Conversely, grapeseed oil, tallow, sunflower oil, and peanut oil exhibited minimal omega-3 contents of < 0.005 g/g. These results are consistent with prior research that emphasized the limited roles of these oils in providing essential omega-3 FAs and highlights flaxseed oil as a superior source of ALA for human health (2, 17).

**Table 1:** Fat contents and omega-3 levels in edible oils.

**Edible oil**	**Omega-3 in fat (%)**	**Fat content (%)**	**Omega-3 (g/g)**
Flaxseed oil	57.7	92.8	0.54
Walnut oil	13.6	99.9	0.14
Rapeseed oil	10.1	99.90	0.10
Canola oil	7.66	99.9	0.08
Soybean oil	6.82	100.0	0.07
Corn oil	2.72	99.8	0.03
Rice bran oil	1.86	99.9	0.02
White sesame oil	1.68	100.0	0.02
Chicken oil	1.48	99.8	0.01
Lard	1.04	99.7	0.01
Palm kernel oil	0.97	99.9	0.01
Butter	1.06	82.7	0.01
Sesame oil: black	0.78	99.7	0.01
Olive oil	0.66	100.0	0.01
Camellia oil	0.61	99.9	0.01
Safflower oil	0.52	100.0	0.01
Safflower oil (high oleic oil)	0.51	99.6	0.01
Sunflower seed oil	0.51	100.0	0.01
Grapeseed oil	0.34	100.00	< 0.01
Tallow	0.31	99.10	< 0.01
Pumpkin seed oil	0.30	99.90	< 0.01
Sunflower seed oil (high oleic acid)	0.12	99.90	< 0.01
Peanut oil	0.09	99.90	< 0.01

**Figure 1:**
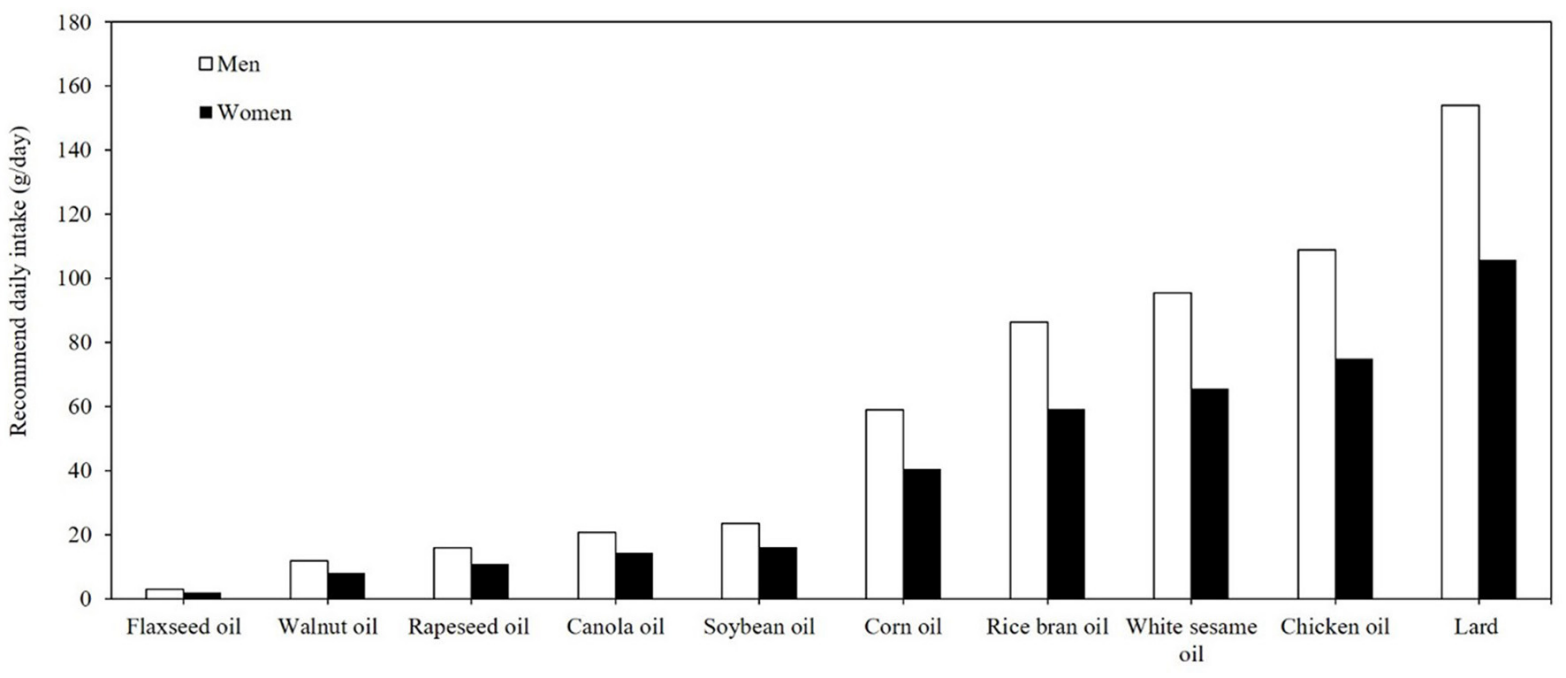
Daily minimal required consumption (g/day) of the top 10 edible oils to meet recommended ALA intake for adults.

Figure 1 illustrates the ranking of the top 10 oils based on their omega-3 FA contents, specifically ALA. The results enabled the quantification of the daily minimally required consumption (g/g) of each oil for adults, providing a clear representation of each item's contribution to the recommended daily ALA intake level. According to recommended oil intake guidelines issued by the Taiwanese Ministry of Health and Welfare, the suggested daily oil intake is 20%−30% of total calories (20). For a 70 kg adult male, the recommended daily fat intake is 70 g, and for a 55 kg adult female, the recommended daily fat intake is 55 g (20). From this perspective, flaxseed oil, rapeseed oil, walnut oil, rapeseed oil, canola oil, soybean oil, and corn oil can fulfill the daily ALA requirement set by the NIH within practical consumption limits, assuming all oil intake comes from the above-mentioned sources (12).

Moreover, considering ~15% conversion rate from ALA to EPA and DHA (17, 20), consuming flaxseed oil, rapeseed oil, walnut oil, canola oil, and soybean oil to meet the recommended daily oil intake can satisfy the 250 mg recommendation of EPA and DHA set by the WHO (2023) and European Food Safety Authority (EFSA) (2012) (1, 14, 21). These findings are particularly relevant for populations transitioning to plant-based diets or reducing marine-based omega-3 sources. Supplementation with flaxseed oil is the most efficient way to boost omega-3 intake. As cold-pressed flaxseed oil is prone to oxidation at high temperatures, it is recommended to consume flaxseed oil raw, either as a drink or in cold dishes, to preserve its nutritional benefits.

However, it was reported that as temperatures increase due to climate-based variations, ALA concentrations in soybeans may significantly decrease from 13 to 3.5%, potentially resulting in inadequate daily ALA intake (21). Additionally, dietary oil sources often include lipids from meats, nuts, and other foods, making it less feasible to rely solely on soybean oil for sufficient omega-3 intake. Canola oil and rapeseed oil are better alternatives, as their FA compositions contain higher ALA and are primarily oleic acid (C18:1), which is relatively stable during cooking.

### 3.2 Seeds and nuts

Table 2 presents omega-3 FA compositions of various nuts and seeds, with flaxseed, chia seed, and awkeotsang (*Ficus pumilla*) seed exhibiting the highest omega-3 concentrations, respectively containing 0.22, 0.20, and 0.08 g/g. Walnuts were also identified as a significant source, providing 0.07 g/g of omega-3. The top three sources of omega-3 had proportions exceeding 50%, with walnuts containing 10.5%. In contrast, commonly consumed nuts, such as pistachios, almonds, macadamia nuts, hazelnuts, and cashews, had significantly lower omega-3 ratios, not exceeding 1%, with omega-3 contents averaging < 0.01 g/g. A similar ranking of edible oils and fats based on omega-3 contents was observed, except for awkeotsang seed and chia seed. Awkeotsang seeds, traditionally used for their rich pectin content in dessert preparations, have potential as an emerging omega-3 oil source. In contrast, chia seeds, due to their mucilage-coated surface, yield less stable oil when pressed, making direct consumption of chia flour more convenient than using the oil form (22–24).

**Table 2:** Fat contents and omega-3 levels in seeds and nuts.

**Nuts and seeds**	**Omega-3 in fat (%)**	**Fat content (%)**	**Omega-3 (g/g)**
Flaxseed	54.0	40.3	0.22
Chia seed	63.0	31.8	0.20
Awkeotsang	62.8	12.5	0.08
Walnut	10.5	67.9	0.07
Pistachio	0.4	52.7	< 0.01
White sesame	0.3	58.7	< 0.01
Black sesame	0.3	54.4	< 0.01
Macadamia nut	0.2	71.6	< 0.01
Pumpkin seed	0.3	47.9	< 0.01
Sunflower seed	0.2	51.9	< 0.01
Hazelnut	0.1	66.5	< 0.01
Cashew nut	0.2	43.7	< 0.01
Watermelon seed	0.2	37.7	< 0.01
Lotus nut	11.4	0.5	< 0.01
Peanut	0.1	43.9	< 0.01
Chestnut	6.2	0.8	< 0.01
Gingko nut	2.3	1.2	< 0.01
Almond	< 0.05	49.8	< 0.01
Pine nut	0.2	69	< 0.01

**Figure 2:**
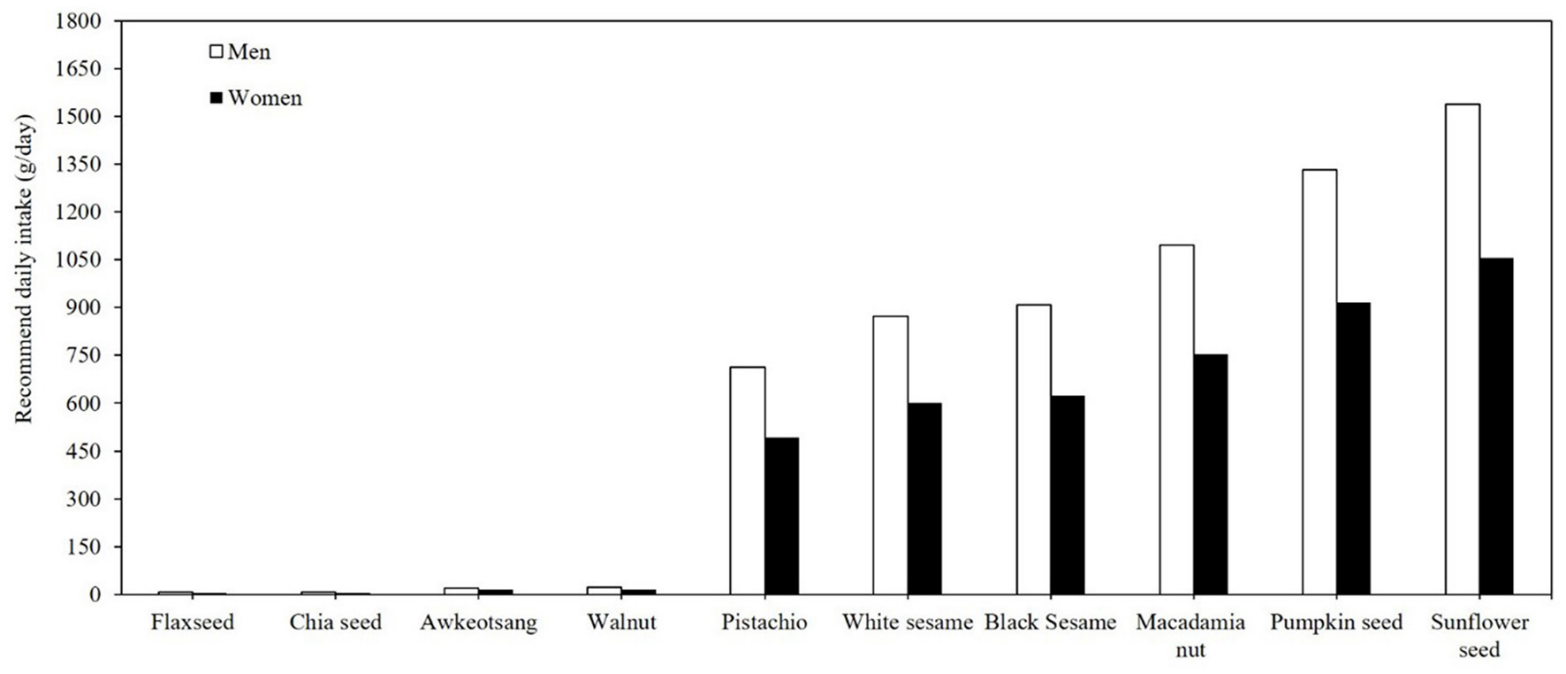
Daily minimal required consumption (g/day) of the top 10 nuts and seeds to meet recommended ALA intake for adults.

Figure 2 illustrates the ranking of the top 10 seeds and nuts recommended for daily consumption in Taiwan. The data underscore the nutritional superiority of flax seed and chia seed as primary plant-based sources of ALA, aligning with previous studies that highlighted their role in omega-3 supplementation (3). Incorporating 5 g and 7 g of flaxseed or chia seed or consuming 5 and 7 walnuts in the daily diet of women and men, respectively, would be adequate to meet the recommended daily ALA intake (17). Considering an ~15% conversion rate from ALA to EPA and DHA (17), consuming flaxseed, chia seed, and walnuts in the a forementioned quantities would fulfill the recommended daily intake of the 250 mg EPA and DHA requirement set by the WHO (2023) and EFSA (2012) (13, 14). These findings suggest that these nuts and seeds offer a practical solution for individuals seeking to enhance omega-3 intake, particularly those relying on plant-based diets. The low omega-3 contents in other nuts and seeds suggest their limited efficacy as standalone sources for meeting omega-3 requirements. For example, consumption of several hundred grams per day of pistachios and sesame seeds would be required to meet the recommended ALA intake.

### 3.3 Fish species

In Table 3, omega-3 FA concentrations across various aquatic species are identified and analyzed, highlighting their potential contributions to daily nutritional requirements. Among fish species, those with omega-3 contents of >0.02 g/g included mackerel (*Scomber scombrus*) (0.08 g/g), Pacific saury (*Cololabis saira*) (0.05 g/g), and starry butterfish (*Stromateus stellatus*) (0.03 g/g). Atlantic salmon (*Salmo salar*), shishamo (*Spirinchus lanceolatus*), Antarctic toothfish (*Dissostichus eleginoides*), fresh-water eel (*Anguilla japonica*), croaker (*Larimichthys polyactis*), runner *(Rachycentron canadum*), silverfish (*Trichiurus japonicus*), spotted tangingi (*Scomberomorus guttatus*), skipjack (*Katsuwonus pelamis*), and flounder (*Pseudorhombus arsius*) were also rich dietary sources of EPA and DHA, with omega-3 contents of 0.02 g/g.

**Table 3:** Fat contents and omega-3 levels in fish.

**Fish species**	**Omega-3 in fat (%)**	**Fat content (%)**	**Omega-3 (g/g)**
Mackerel (*Scomber scombrus*)	20.5	39.4	0.08
Pacific saury (*Cololabis saira*)	21.0	21.8	0.05
Starry butterfish (*Stromateus stellatus*)	18.8	16.3	0.03
Atlantic salmon (*Salmo salar*)^a^	16.7	14.9	0.02
Shishamo (*Spirinchus lanceolatus*)	47.6	4.9	0.02
Antarctic toothfish (*Dissostichus eleginoides*)	8.9	25.5	0.02
Freshwater eel (*Anguilla japonica*)	11.3	19.6	0.02
Croaker (*Larimichthys polyactis*)	22.8	8.0	0.02
Runner (*Rachycentron canadum*)	15.1	7.2	0.02
Silverfish (*Trichiurus japonicus*)	18.3	2.0	0.02
Spotted tangingi (*Scomberomorus guttatus*)	15.1	10.4	0.02
Skipjack (*Katsuwonus pelamis*)	27.7	5.6	0.02
Flounder (*Pseudorhombus arsius*)	9.5	16.1	0.02
Croceine croaker (*Larimichthys crocea*)	17.7	7.8	0.01
Yellow croaker [*Larimichthys croceus* (Richardson)]	15.7	1.6	0.01
East Asian fourfinger threadfin (*Eleutheronema rhadinum*)	17.5	7.2	0.01
Spaniard (*Scomberomorus commerson*)	11.9	10.4	0.01
Thornfish (*Terapon jarbua*)	14.0	8.2	0.01
Chicken grunt (*Parapristipoma trilineatum*)	20.6	5.4	0.01
Rainbow trout (*Oncorhynchus mykiss*)	16.1	6.8	0.01
Japanese butterfish (*Psenopsis anomala*)	12.4	8.7	0.01
Southern mackerel (*Scomber australasicus*)	26.5	4.0	0.01
Sliver stripe round herring (*Spratelloides gracilis*)	75.5	1.3	0.01
Black sea bream (*Acanthopagrus schlegelii*)	10.5	9.0	0.01
Blackspot barracuda (*Sphyraena forsteri*)	13.2	7.1	0.01
Largemouth black bass (*Micropterus salmoides*)	19.0	4.6	0.01
Striped bass (*Morone saxatilis*)	13.9	6.2	0.01
Razor trevally (*Mene maculata*)	29.4	2.9	0.01
Scat (*Scatophagus argus)*	6.5	12.6	0.01
Big head (*Hypophthalmichthys nobilis*)	11.0	7.2	0.01
Yellow fin seabream (*Acanthopagrus latus*)	13.2	6.0	0.01
Purplish amberjack (*Seriola dumerili*)	32.3	2.4	0.01
Tiger grouper (*Epinephelus fuscoguttatu*s)	20.7	3.7	0.01
Yellowfin sea bream (*Acanthopagrus latus*)	15.1	4.8	0.01
Rabbitfish (*Siganus fuscescens*)	8.2	8.8	0.01
Round scad (*Decapterus macrosoma*)	28.1	2.4	0.01
Golden thread (*Nemipterus virgatus*)	18.9	3.3	0.01
Japanese jack (*Trachurus japonicus*)	30.9	2.0	0.01
Spottedtail morwong (*Cheilodactylus zonatus*)	18.2	3.4	0.01
Silver sea bream (*Rhabdosargus sarba*)	13.2	4.4	0.01
Silver pomfret (*Pampus argenteus*)	9.9	5.8	0.01
Sand snapper (*Lethrinus nebulosus*)	16.9	3.4	0.01
Lizardfish (*Saurida undosquamis*)	33.1	1.6	0.01
Torpedo scad (*Megalaspis cordyla*)	32.1	1.6	0.01
Tang's snapper (*Lipocheilus carnolabrum*)	13.9	3.7	0.01
Redtail scad (*Decapterus kurroides*)	29.6	1.7	0.01
Ornate surgeonfish (*Acanthurus dussumieri*)	8.1	6.2	0.01
Sea mullet (*Mugil cephalas*)	5.7	8.8	0.01

^a^Indicates the average value derived from different parts of the Atlantic salmon within that category.

Among the 13 fish species identified with an omega-3 content of at least 0.01 g/g, several are globally recognized for their economic and dietary importance. These include rainbow trout (*Oncorhynchus mykiss*), southern mackerel (*Scomber australasicus*), black sea bream (*Acanthopagrus schlegelii*), largemouth black bass (*Micropterus salmoides*), striped bass (*Morone saxatilis*), silver pomfret (*Pampus argenteus*), sand snapper (*Lethrinus nebulosus*), round scad (*Decapterus macrosoma*), golden thread (*Nemipterus virgatus*), sea mullet *(Mugil cephalas*), and tiger grouper (*Epinephelus fuscoguttatus*). These species are widely distributed across temperate, tropical, and subtropical regions, making them accessible and popular choices for human consumption. Notably, rainbow trout and silver pomfret are highly valued in both Western and Asian cuisines for their rich flavor and nutritional benefits, while southern mackerel and black sea bream are prominent in Asian markets for their affordability and high omega-3 contents. Largemouth black bass and striped bass hold significant importance in recreational fishing and aquaculture sectors worldwide. Additionally, golden thread, round scad, and sand snapper are essential to commercial fisheries, particularly in Southeast Asia, where they are integral to regional diets. The global presence and widespread consumption of these fish species highlight their critical role in providing omega-3 FAs, significantly contributing to cardiovascular and neurological health when included as part of a balanced diet.

The consumption of 48 fish species with omega-3 contents of ≥0.01 g/g at the recommended daily amount of 1.1 ounces (oz.; 34.2 g) can meet and exceed the suggested omega-3 intake (250 mg/day) for cardiovascular and neurological health. This confirms that adhering to the US dietary guideline of consuming 8 oz. of fish per week is sufficient to fulfill the omega-3 recommendation when selecting fish species with an omega-3 content of ≥0.01 g/g. These findings emphasize the nutritional importance of incorporating these fish species into regular diets to ensure adequate omega-3 intake.

**Figure 3:**
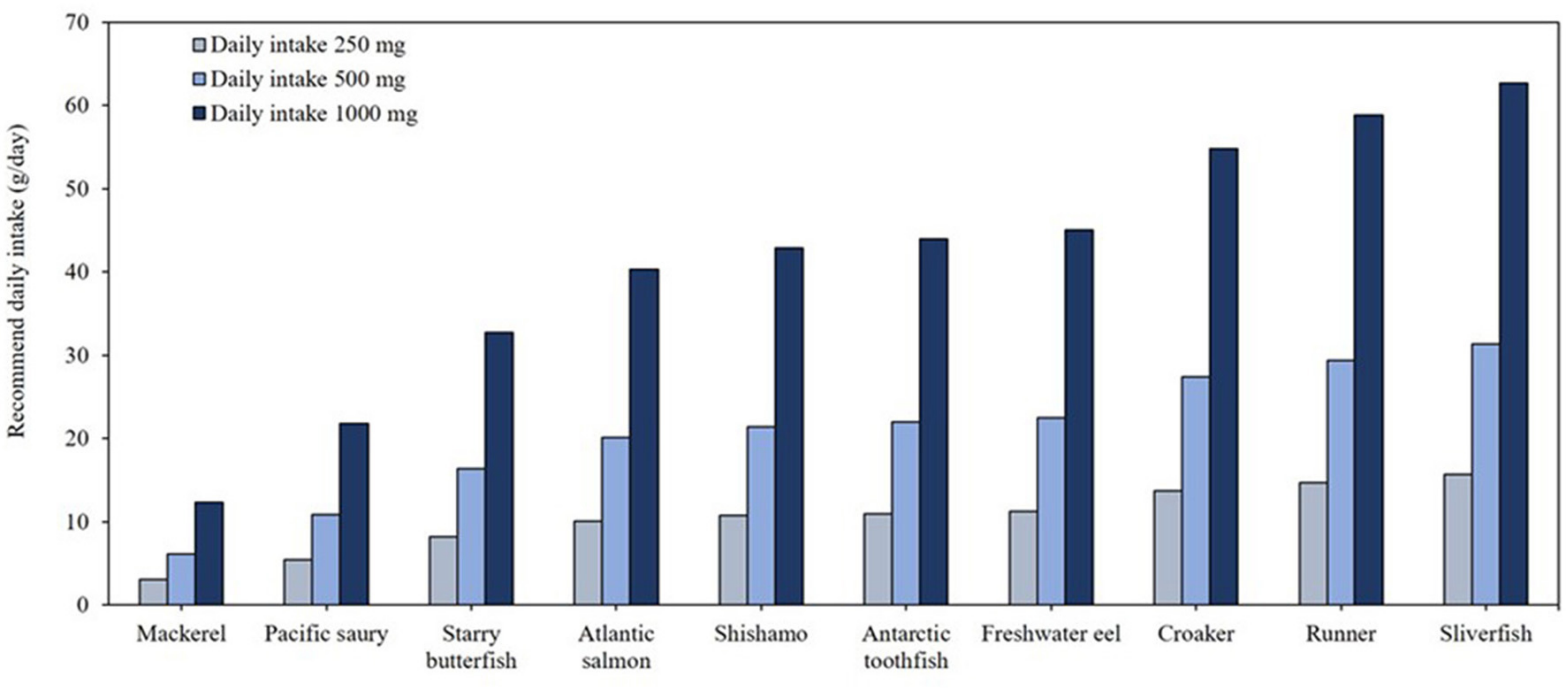
Daily minimal required consumption (g/day) of the top 10 fish species to meet recommended EPA and DHA intake levels.

Figure 3 highlights the top 10 fish species with the highest omega-3 contents, demonstrating their efficiency in meeting daily omega-3 intake requirements. To achieve the recommended 250 mg/day of omega-3, consumption of ~10 g of these high omega-3 species is sufficient. For individuals targeting higher intake levels, such as 500 mg/day, consuming 6–31 g of fish is adequate, while for 1,000 mg/day, the required intake ranges 12–63 g depending on the specific fish species. Given the US dietary guideline recommendation of consuming 31.2 g/day (1.1 oz.) of fish, selecting these high omega-3 fish species can effectively enhance dietary omega-3 intake (15).

Supplementary Table 1A lists 83 fish species with omega-3 contents of < 0.01 g/g, many of which are still widely consumed globally. Common species in this category include red seabream (*Pagrus major*), anchovy (*Encrasicholina heteroloba*), Nile tilapia (*Oreochromis niloticus*), and tuna (*Thunnus sp*.). While these fish provide only limited omega-3 supplementation, they remain valuable dietary components for other health benefits, such as being rich sources of lean protein, essential vitamins (vitamins D and B12), and minerals (selenium and iodine). However, for individuals specifically targeting omega-3 intake, reliance on those species with higher omega-3 contents remains essential.

### 3.4 Mollusk species

Table 4 presents omega-3 contents and crude fat compositions of mollusk species. Among the 15 species analyzed, Argentine shortfin squid (*Illex argentinus*), green mussel (*Perna viridis*), giant Pacific oyster (*Crassostrea gigas*), and yesso scallop (*Patinopecten yessoensis*) exhibited omega-3 concentrations of 0.01 g/g, with crude fat ranging 1.2%−2.2%. As shown in Figure 5, to meet the minimum daily omega-3 recommendation of 250 mg/day, consumption of ~24–49 g of these species is required. For individuals targeting higher intake levels, such as 500 mg/day, consuming 48–98 g of these mollusks would suffice, while for 1,000 mg/day, the required daily intake ranges 96–97 g. Mollusks with < 0.01 g/g omega-3 contents such as corbicula clam (*Corbicula fluminea*) and Chinese Venus (*Cyclina sinensis*) would contribute minimally to omega-3 supplementation.

**Table 4:** Fat contents and omega-3 levels in mollusks.

**Mollusk species**	**Omega-3 in fat (%)**	**Fat content (%)**	**Omega-3 (g/g)**
Argentine shortfin squid (*Illex argentinus*)	52.2	2.0	0.01
Green mussel (*Perna viridis*)	46.4	2.2	0.01
Giant Pacific oyster (*Crassostrea gigas*)	37.9	1.6	0.01
Yesso scallop (*Patinopecten yessoensis*)	42.4	1.2	0.01
Diphos sanguine (*Sanguinolaria diphos*)	26.7	1.6	< 0.01
Bigfin reef squid (*Sepioteuthis lessoniana*)	42.2	0.8	< 0.01
Corbicula clam (*Corbicula fluminea*)	18.4	1.4	< 0.01
Undulating Venus (*Paphia undulata*)	25.7	1.0	< 0.01
Chinese Venus (*Cyclina sinensis*)	24.5	1.0	< 0.01
Inshore squid (*Loligo edulis*)	41.3	0.4	< 0.01
Scallop (*Patinopecten yessoensis*)	37.5	0.4	< 0.01
Cuttlefish (*Sepia esculenta*)	46.4	0.3	< 0.01
Filipino Venus (*Ruditapes philippinarum*)	24.6	0.5	< 0.01
Abalone (*Haliotis diversicolor*)	66.7	0.1	< 0.01
Jackknife clam (*Solen strictus*)	15.7	0.3	< 0.01

Table 5 summarizes the omega-3 content distribution across edible oils, nuts/seeds, fish, crustaceans, and mollusks, categorized by concentration levels. Among edible oils, 22.7% of the samples, including flaxseed oil and walnut oil, exhibited omega-3 concentrations exceeding 0.02 g/g, making them the most efficient sources of ALA, while 54.6% had moderate levels (0.01–0.02 g/g). In contrast, 83.3% of nuts and seeds contained omega-3 levels of < 0.01 g/g, with only flaxseed, chia seed, and awkeotsang demonstrating concentrations above 0.2 g/g. For fish, 9.9% of species, such as mackerel, Pacific saury, and starry butterfish, had omega-3 concentrations exceeding 0.02 g/g, while 34.3% fell within a moderate range (0.01–0.02 g/g), and the majority (63.4%) had levels of < 0.01 g/g, underscoring the importance of fatty fish as efficient sources of EPA and DHA. Among crustaceans, 19.0% of species, including northern shrimp, Hokkai shrimp, and crab, contained omega-3 levels of 0.01 g/g, while 81.0% exhibited lower concentrations. Similarly, 26.7% of mollusks, such as Argentine shortfin squid and green mussel, had moderate omega-3 contents (0.01 g/g), with the remaining 73.3% below this threshold. These findings underscore the efficiency of specific edible oils, seeds, nuts, and fatty fish as significant omega-3 sources, while crustaceans and mollusks only provide moderate contributions to daily intake requirements.

**Table 5:** Daily intake of α-linolenic acid (ALA) via edible oil, nuts and seeds, and all foods in Taiwan.

**Age (year)**	**ALA intake (g/day)**																	
	**Edible oils**						**Nuts and seeds**						**All foods** ^a^					
	**Men**			**Women**			**Men**			**Women**			**Men**			**Women**		
	**Mean**	**P50**	**P95**	**Mean**	**P50**	**P95**	**Mean**	**P50**	**P95**	**Mean**	**P50**	**P95**	**Mean**	**P50**	**P95**	**Mean**	**P50**	**P95**
0–3	0.202	0.041	0.563	0.203	0.042	0.565	0.899	0.132	5.352	0.854	0.126	5.082	1.102^#^	0.174	5.914^#^	1.057^#^	0.167	5.647^#^
3–6	0.473	0.097	1.315	0.421	0.086	1.172	1.738	0.256	10.339	1.722	0.254	10.245	2.210^#^	0.353	11.654^#^	2.143^#^	0.340	11.417^#^
6–12	0.722	0.148	2.006	0.594	0.122	1.653	2.125	0.313	12.647	1.894	0.279	11.270	2.847^#^	0.461	14.654^#^	2.488^#^	0.400	12.923^#^
12–16	0.951	0.194	2.644	0.728	0.149	2.025	2.176	0.321	12.949	2.176	0.321	12.949	3.127^#^	0.515	15.592^#^	2.904^#^	0.469	14.974^#^
16–18	1.004	0.205	2.792	0.711	0.145	1.978	2.965	0.437	17.641	1.847	0.272	10.988	3.969^#^	0.642	20.433^#^	2.558^#^	0.417	12.966^#^
19–65	0.856	0.175	2.381	0.711	0.145	1.978	3.342	0.492	19.888	2.738	0.403	16.293	4.199^#^	0.667	22.269^#^	3.449^#^	0.549	18.270^#^
>65	0.672	0.137	1.869	0.551	0.113	1.533	2.602	0.383	15.481	2.307	0.340	13.726	3.274^#^	0.521	17.349^#^	2.858^#^	0.453	15.260^#^

^a^All foods are calculated as the total of edible oils, seeds, and nuts. P50, 50th percentile.

Number sign (#): The daily intake of omega-3 exceed adequate intake (AI) of National Institutes of Health (NIH) recommendation: infants aged 0–6 months, 0.32 g/day; infants aged 7–12 months, 0.5 g/day; children aged 1–3 years, 0.7 g/day; children aged 4–8, 0.9 g/day; boys aged 9–13 years, 1.2 g/day; girls aged 9–13 years, 1.0 g/day; boys aged 14–18 years and men aged 19+ years, 1.6 g/day; girls aged 14–18 years and women aged 19+ years, 1.1 g/day (17).

### 3.5 Crustacean species

**Figure 4:**
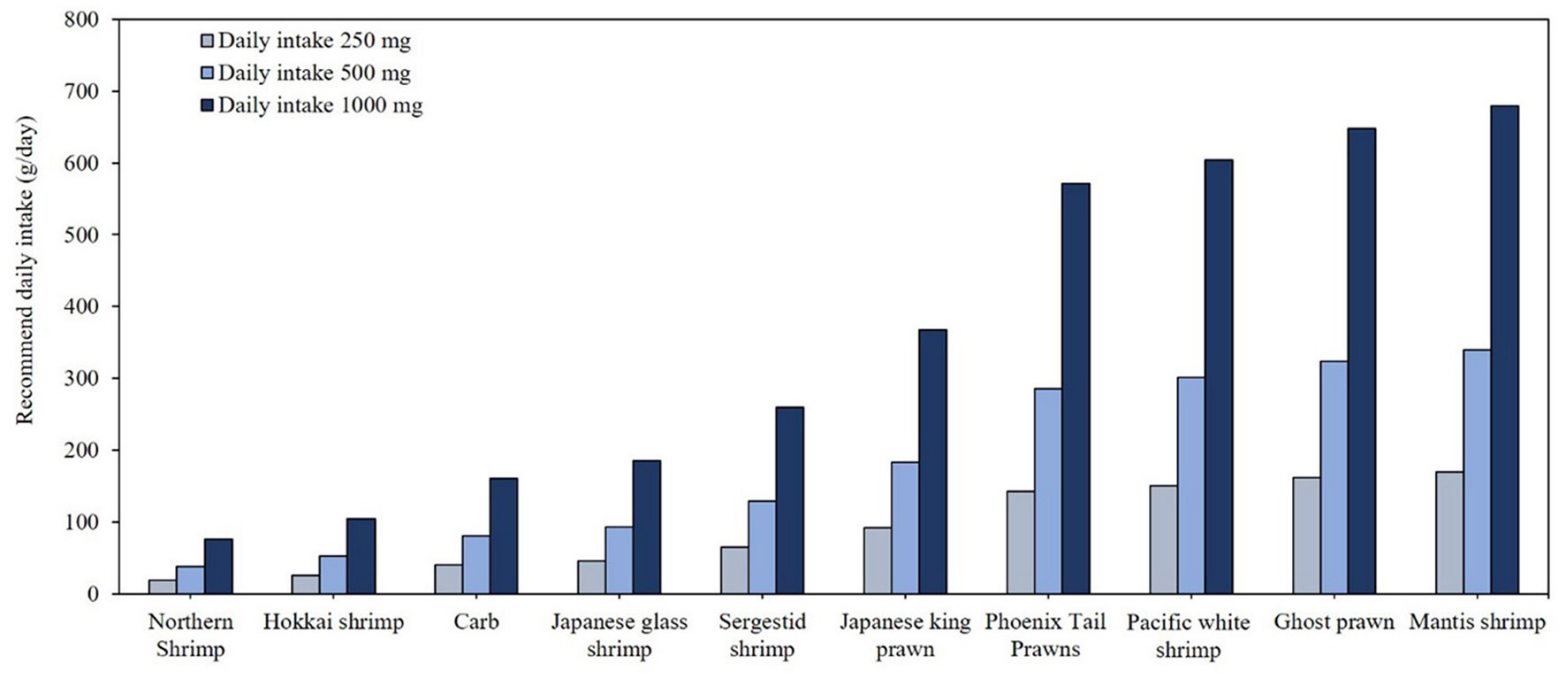
Daily minimal required consumption (g/day) of the top 10 crustacean species to meet recommended EPA and DHA intake levels.

Table 6 presents the omega-3 contents and fat contents of various crustacean species. Among the 21 crustacean species analyzed, only four species, northern shrimp (*Pandalus borealis*), Hokkai shrimp (*Aristaeomorpha foliacea*), crab (*Scylla serrata*), and Japanese glass shrimp (*Pasiphaea japonica*), demonstrated omega-3 concentrations of 0.01 g/g, with crude fat ranging 1.5%−6.1%. According to Figure 4, these species could modestly contribute to daily omega-3 intake and require consumption of ~19–46 g/day to meet the minimum omega-3 recommendation of 250 mg/day. The top 10 species consist of various types of shrimp and prawn, with only one crab species included. To meet the 500 mg omega-3 intake requirement, consumption of 38–340 g is necessary, while achieving 1,000 mg would require twice that amount.

**Table 6:** Fat contents and omega-3 levels in crustaceans.

**Crustacean species**	**Omega-3 in fat (%)**	**Fat content (%)**	**Omega-3 (g/g)**
Northern shrimp (*Pandalus borealis*)	32.0	4.1	0.01
Hokkai shrimp (*Aristaeomorpha foliacea*)	15.7	6.1	0.01
Crab (*Scylla serrata*)	17.2	3.6	0.01
Japanese glass shrimp (*Pasiphaea japonica*)	35.9	1.5	0.01
Sergestid shrimp (*Sergia lucens*)	29.7	1.3	< 0.01
Japanese king prawn (*Marsupenaeus japonicus*)	38.9	0.8	< 0.01
Phoenix tail prawn (*Penaeus vannamei*)	21.9	0.8	< 0.01
Pacific white shrimp (*Litopenaeus vannamei*)	18.4	0.9	< 0.01
Ghost prawn (*Palaemonetes paludosus*)	22.1	0.7	< 0.01
Mantis shrimp (*Gonodactylus smithii*)	29.4	0.5	< 0.01
Whiskered velvet shrimp (*Metapenaeopsis barbata*)	36.3	0.4	< 0.01
Blue shrimp (*Litopenaeus stylirostris*)	23.5	0.6	< 0.01
Sagami lobster (*Metanephrops sagamiensis*)	43.1	0.3	< 0.01
Big head prawn (*Solenocera crassicornis*)	30.0	0.3	< 0.01
Armored lobster (*Heterocarpus sibogae*)	27.8	0.3	< 0.01
Hard spear prawn (*Parapenaeopsis hardwickii*)	32.2	0.2	< 0.01
Big head prawn (*Solenocera alticarinata*)	26.3	0.2	< 0.01
Kuruma shrimp (*Penaeus chinensis*)	25.8	0.2	< 0.01
Japanese spiny lobster (*Panulirus japonicus*)	46.7	0.1	< 0.01
Crimson crab (*Ranina ranina*)	43.7	0.1	< 0.01
Giant river shrimp (*Macrobrachium rosenbergii*)	18.0	0.1	< 0.01

Despite majority of crustacean species exhibiting omega-3 concentrations below 0.01 g/g, they remain valuable dietary options due to their rich nutritional profiles, which include lean protein, essential minerals (zinc, selenium, and copper), and low saturated fat contents. Species such as Japanese king prawn (*Marsupenaeus japonicus*), kuruma shrimp (*Penaeus chinensis*), and Sagami lobster (*Metanephrops sagamiensis*) are commonly consumed but have omega-3 levels too low to significantly contribute to daily requirements, highlighting their limited role as primary omega-3 sources (Table 6).

### 3.6 Evaluation of ALA, EPA, and DHA daily intake adequacy

This study assessed the adequacy of daily intake of ALA, EPA, and DHA using dietary data from the Food Consumption Database of the Taiwan Food and Drug Administration (19). Intake estimates were stratified by age and gender. Tables 5, 7 present the daily intake of ALA, EPA, and DHA from five major food categories among Taiwanese males and females across various age groups. The results indicate that ALA intake from seeds and nuts was ~2.3–4.4 times higher than that from edible fats and oils, based on mean intake values. In the P95 intake group, ALA consumption from nuts and seeds was nearly 10 times greater than that from edible oils (Table 5). For DHA and EPA, intake followed the descending order of fish > crustaceans > mollusks (Table 7). No significant gender differences in daily intake were observed.

**Table 7:** Daily intake of eicosapentaenoic acid (EPA) and docosahexaenoic acid (DHA) by respondents in Taiwan.

**Age (year)**	**EPA and DHA intake (g/day)**																							
	**Fish**						**Crustaceans**						**Mollusks**						**All foods** ^a^					
	**Men**			**Women**			**Men**			**Women**			**Men**			**Women**			**Men**			**Women**		
	**Mean**	**P50**	**P95**	**Mean**	**P50**	**P95**	**Mean**	**P50**	**P95**	**Mean**	**P50**	**P95**	**Mean**	**P50**	**P95**	**Mean**	**P50**	**P95**	**Mean**	**P50**	**P95**	**Mean**	**P50**	**P95**
0–3	0.152	0.129	0.258#	0.100	0.085	0.170	0.025	0.021	0.042	0.017	0.014	0.028	0.002	0.002	0.004	0.005	0.004	0.007	0.180	0.152	0.304#	0.121	0.103	0.205
3–6	0.146	0.124	0.248	0.154	0.130	0.261#	0.035	0.029	0.058	0.027	0.022	0.045	0.011	0.009	0.018	0.004	0.003	0.006	0.192	0.162	0.325#	0.184	0.156	0.312#
6–12	0.157	0.134	0.267#	0.169	0.144	0.287#	0.035	0.029	0.059	0.036	0.030	0.060	0.027	0.021	0.042	0.009	0.007	0.014	0.219	0.184	0.369#	0.214	0.181	0.362#
12–16	0.165	0.141	0.281#	0.160	0.136	0.273#	0.024	0.020	0.040	0.030	0.025	0.050	0.006	0.005	0.009	0.008	0.006	0.012	0.195	0.165	0.330#	0.198	0.167	0.335#
16–18	0.175	0.149	0.298#	0.076	0.064	0.129	0.032	0.027	0.054	0.027	0.023	0.046	0.006	0.005	0.010	0.012	0.009	0.018	0.214	0.181	0.362#	0.114	0.096	0.192
19–65	0.332#	0.282#	0.565#	0.271#	0.230	0.461#	0.038	0.032	0.064	0.031	0.026	0.051	0.016	0.012	0.025	0.013	0.010	0.020	0.386#	0.327#	0.654#	0.315#	0.266#	0.533#
>65	0.558#	0.474#	0.949#	0.360#	0.306#	0.612#	0.020	0.017	0.034	0.015	0.012	0.025	0.013	0.010	0.021	0.010	0.008	0.015	0.592#	0.502#	1.004#	0.385#	0.326#	0.652#

^a^All foods are calculated as the total of fish, crustaceans, and mollusks. P50, 50th percentile.

Number sign (#): The daily intake of omega-3 exceeds adequate intake (AI) of the World Health Organization (WHO) recommendation (0.25–0.5 g/day) (13).

Among males, the total ALA intake from edible oils, nuts, and seeds ranged 1.102–4.199 g/day (mean), 0.174–0.667 g/day at P50 (50th percentile), and 5.914–22.269 g/day at P95. For females, mean ALA intake ranged 1.057–3.449 g/day, with P50 values of 0.167–0.549 g/day and P95 values ranging 5.647–18.270 g/day. When examining individual food categories, both males and females exhibited mean, P50, and P95 omega-3 intake levels below recommended levels established by the NIH (17). However, when the total ALA intake from fats, nuts, and seeds was considered, both the mean and P95 values exceeded NIH recommendations, whereas P50 values remained below recommended levels.

Regarding EPA and DHA intake from fish, crustaceans, and mollusks, male intake ranged 0.180–0.592 g/day (mean), 0.152–0.502 g/day at P50, and 0.304–1.004 g/day at P95. Among females, mean DHA and EPA intake ranged 0.114–0.384 g/day, with P50 values of 0.096–0.326 g/day and P95 values ranging 0.192–0.652 g/day. For EPA and DHA, the mean and P50 intake levels from fish among males were below the recommended intake of 250 mg/day, except for those older than 19 years. In contrast, females demonstrated greater inadequacies, with mean intake among those younger than 18 years consistently falling below the recommended intake, and P50 intake meeting the recommended levels only in elderly populations (>65 years). When total EPA and DHA intake from fish, crustaceans, and mollusks was considered, the mean and P50 daily intake levels among adults and the elderly fell within the NIH recommended range. However, at P95, DHA and EPA intake was sufficient, whereas adolescent girls (16–18 years) and female children under 3 years failed to meet recommended intake levels.

These findings suggest that gender had minimal influence on overall omega-3 intake, while age appeared to play a more significant role in determining intake adequacy. They also highlight the need for targeted dietary interventions to improve omega-3 intake, particularly for vulnerable age group.

## 4 Discussion

As is well known, the daily amount of aquatic species required to meet omega-3 intake needs largely depends on their omega-3 concentration. Species with higher omega-3 contents reduce the necessary intake volume, making them more efficient dietary sources. This underscores the importance of incorporating a diverse range of fish, crustaceans, and mollusk into one's daily diet to optimize health. Notably, further investigations are warranted to assess the impacts of cooking methods and food processing on omega-3 bioavailability, as well as to promote the sustainable management of high demand aquatic species to mitigate environmental impacts (25, 26). Previous studies on red mullet (*Mullus barbatus*) have shown that cooking methods markedly influence lipid content and fatty acid composition. For example, frying and microwaving significantly increased total lipids but reduced EPA and DHA proportions, whereas grilling and boiling preserved essential n-3 fatty acids, indicating that choice of cooking method can substantially affect the nutritional quality of seafood (27).

The study also compared the TFDA nutrition database for edible oils (Figure 1) and seeds and nuts (Figure 2) with the US Department of Agriculture (USDA) rankings of ALA-rich foods. In line with USDA data, flaxseed oil emerged as the richest source of ALA, containing ~0.49 g/g, while canola oil provided 0.09 g/g, aligning with global benchmarks (15). However, perilla oil, widely recognized in East Asian diets for its ALA content, was less prominently reported in USDA data, reflecting regional dietary variations. These findings emphasize that flaxseed oil remains the most efficient global source of ALA, while perilla oil serves as a significant regional contributor in East Asia. For seeds and nuts (Figure 2), the TFDA identified flaxseed, chia seed, and walnuts as the top three sources of ALA. This ranking is consistent with USDA data, where flaxseed provides 23.9 g ALA/100 g, followed by chia seeds at 17.8 g/100 g and English walnuts at 9.1 g/100 g (15). These foods not only serve as primary ALA sources but also offer additional nutritional benefits, such as dietary fiber, protein, and antioxidants, which contribute to overall dietary quality and health outcomes.

**Figure 5:**
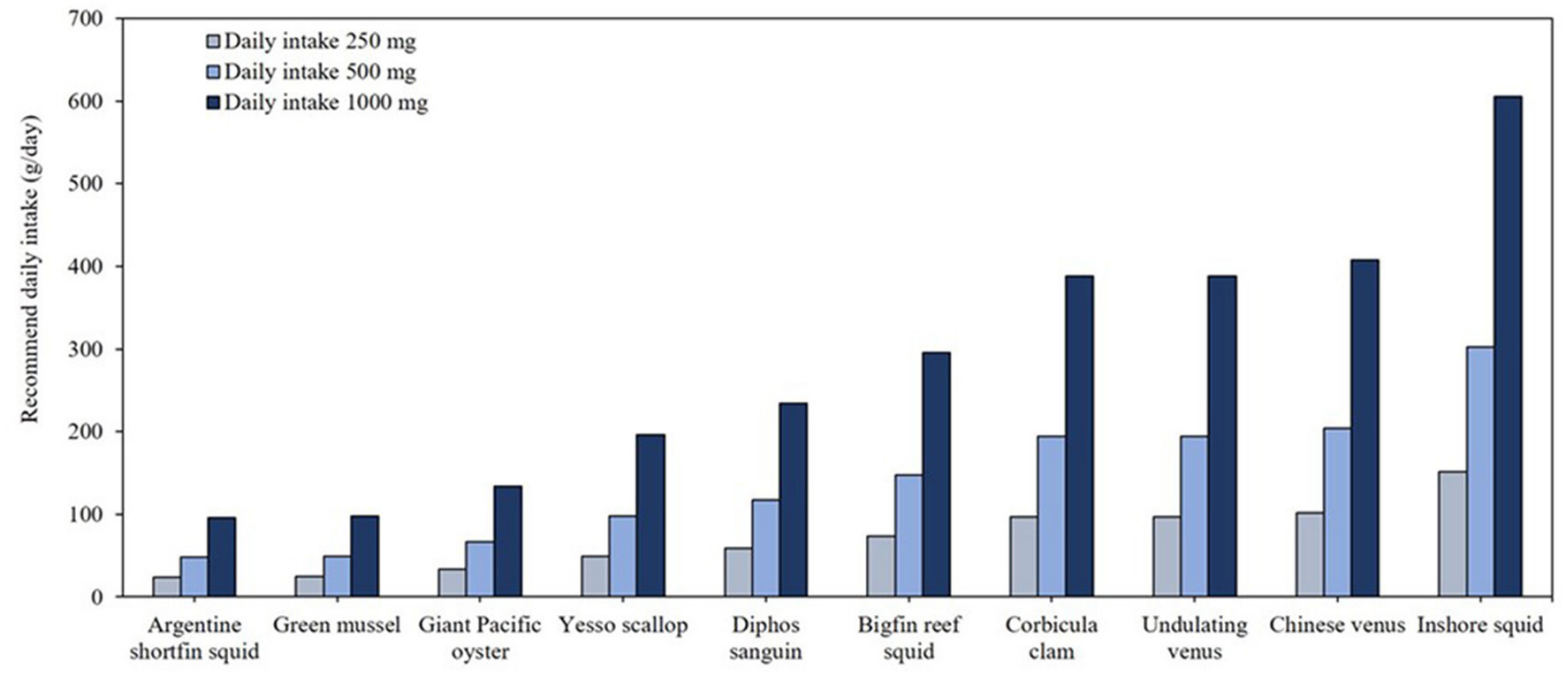
Daily minimal required consumption (g/day) of the top 10 mollusk species to meet recommended EPA and DHA intake levels.

In terms of omega-3 FAs derived from seafood, the study's findings regarding EPA and DHA concentrations showed partial discrepancies compared to USDA data, likely due to differences in fish species and their geographical origins (15). For instance, the fat content of mackerel in this database appears extraordinarily high compared to the USDA database. Previous studies indicated that the fat content of mackerel aligns more closely with that of southern mackerel in the database (28). Atlantic salmon and herring, reported in USDA data to provide 0.02 g/g of combined EPA and DHA (15), demonstrated a similar content in this study, highlighting their exceptional nutritional value. The EPA and DHA contents of Atlantic mackerel, rainbow trout, and sea bass, recorded as ~0.01 g/g in the USDA database, were consistent with findings of this study, confirming their global significance as efficient sources of EPA and DHA. For crustaceans (Figure 5), species such as Hokkai shrimp and crab exhibited moderate omega-3 concentrations of ~0.01 g/g. In contrast, USDA data reported cooked shrimp and lobster as having lower omega-3 contents, typically ranging 0.002–0.003 g/g (15). Mollusks, including green mussels and Argentine shortfin squid, displayed notable omega-3 contents (~0.01 g/g), consistent with USDA findings. For example, eastern oysters were reported to provide ~0.008 g/g of omega-3, emphasizing their nutritional value despite being consumed less frequently compared to fish (15).

The cardiovascular benefits of omega-3 fatty acids, particularly EPA and DHA, are well documented, including their roles in reducing inflammation, lowering triglycerides, and improving cardiovascular outcomes (12). Despite these benefits, no official recommended dietary intake exists for EPA and DHA due to several challenges: insufficient long term clinical trials to define precise requirements, individual variability in physiology and dietary habits, cultural differences in dietary sources, low conversion efficiency of ALA to EPA and DHA, evolving health recommendations, and the clinical focus on specific conditions rather than general population needs. Unlike the essential FA ALA, which has a defined recommended intake for comparison, the daily intake of EPA and DHA in this study was evaluated based on the WHO recommendation of 250–500 mg/day (13). However, different international health organizations propose higher recommended intake levels, such as 1 g/day. The findings of this study indicate that many age groups did not achieve these intake recommendations, suggesting that omega-3 consumption requires further improvement to support optimal health. Referencing international health claims can help raise awareness of omega-3 insufficiency. For example, the U.S. FDA allows a qualified claim that consumption of EPA and DHA may reduce the risk of coronary heart disease, with a maximum recommended intake of 2 g/day (29). Similarly, the EU permits the claim that EPA and DHA contribute to normal heart function for products providing at least 250 mg/day (30). Currently, Taiwan does not have comparable health claims or labeling regulations specifically aimed at promoting omega-3 intake. By following international examples, Taiwan could develop a scientific framework for omega-3 health claims and enhance public communication, thereby promoting informed dietary choices and helping to close the gap in omega-3 intake across the population.

This study is the first to investigate omega-3 intake by integrating a nutrient composition database with a dietary consumption database. This model can serve as a novel research approach for assessing the intake of various essential nutrients. One limitation of this study is that the assessment did not account for omega-3 intake from dietary supplements, which may have led to an underestimation of actual intake levels. Future research should incorporate additional dietary sources, such as meat, algae, and vegetables, to provide a more comprehensive assessment of omega-3 intake in the Taiwanese population.

This study has several limitations. The Food Nutrient Database used in the analysis contained predominantly single entry records for most food items, which precluded the calculation of standard deviations and prevented a robust assessment of variability in nutrient content. Second, differences in the bioavailability of omega-3 fatty acids among various food types were not considered; for the purposes of this analysis, all omega-3 values were treated as being 100% bioavailable. Future studies should incorporate data with higher sampling resolution and address differential bioavailability to provide more accurate dietary recommendations.

## 5 Conclusions

This study highlights the importance of omega-3 fatty acids in the Taiwanese diet and systematically identifies the major contributors of intake from both plant- and animal-based foods. Among plant-derived sources, flaxseed oil, chia seeds, and walnuts were particularly efficient in providing ALA, serving as suitable alternatives for individuals with limited access to marine foods or those following vegetarian diets. In contrast, fatty fish such as mackerel and Pacific saury provided concentrated amounts of EPA and DHA, whereas most crustaceans and mollusks contained relatively low levels, requiring higher consumption volumes to achieve meaningful nutritional benefits. Notably, while certain population groups achieved adequate ALA intake, EPA and DHA intake remained insufficient among younger age groups, especially adolescents and young children. These findings emphasize the need to enhance public awareness regarding omega-3 insufficiency and promote dietary practices that prioritize omega-3 rich foods, thereby supporting healthier long-term nutrition patterns across the Taiwanese population.

## Data Availability

The original contributions presented in the study are included in the article/Supplementary material, further inquiries can be directed to the corresponding author/s.
