# Supplementary material for: Dietary strategies for optimizing omega-3 fatty acid intake: a nutrient database-based evaluation in Taiwan
Source: Front Nutr. 2025 Sep 19;12:1661702. doi: 10.3389/fnut.2025.1661702 (PMC12491008; doi:10.3389/fnut.2025.1661702)
Supplement: Supplementary file 1 [file Table_1.docx]

Supplementary Material

**Table A1.** A detailed list of various types of fish with omega-3 content less than 0.01 g/g.

| Fish type | Omega-3 in fish (%) | Crude fat of fish (%) | Omega-3 (g/g) |
| --- | --- | --- | --- |
| Bulleye **(*Pagrus major*)** | 32.7 | 1.5 | <0.01 |
| Blackfin scad  (*Decapterus maruadsi*) | 22.4 | 2.1 | <0.01 |
| Red seabream (*Pagrus major*) | 18.7 | 2.5 | <0.01 |
| Silver pomfret  (*Monodactylus argenteus*) | 10.5 | 4.4 | <0.01 |
| Compressed mackerel (*Scomberomorus koreanus*) | 11.3 | 4.0 | <0.01 |
| Jaguar guapote  (*Parachromis managuensis*) | 10.9 | 4.1 | <0.01 |
| Seriola rivoliana (*Seriola rivoliana*) | 23.1 | 1.9 | <0.01 |
| Queensland halibut (*Psettodes erumei*) | 3.7 | 11.5 | <0.01 |
| Round scad (*Decapterus maruadsi*) | 32.0 | 1.3 | <0.01 |
| Tiger grouper (*Epinephelus fuscoguttatus*) | 10.7 | 3.7 | <0.01 |
| Whitemargin lyretail grouper (*Variola albimarginata*) | 21.2 | 1.8 | <0.01 |
| Anchovy **(*Encrasicholina heteroloba*)** | 41.6 | 0.9 | <0.01 |
| Triple-tail  (*Lobotes surinamensis*) | 15.6 | 2.3 | <0.01 |
| Rosed razorfish  (*Iniistius verrens*) | 19.9 | 1.8 | <0.01 |
| Palmer (*Lates calcarifer*) | 13.4 | 2.6 | <0.01 |
| Silver fish (*Neosalanx tangkahkeii*) | 37.0 | 0.9 | <0.01 |
| Short nosed sword fish  (*Istiompax indica*) | 30.0 | 1.1 | <0.01 |
| Gadus morrhua  (*Patagonian toothfish*) | 12.9 | 2.5 | <0.01 |
| Striped surgeonfish  (*Acanthurus lineatus*) | 18.8 | 1.7 | <0.01 |
| Bangos (*Chanos chanos*) | 3.4 | 9.5 | <0.01 |
| Japanese rubyfish  (*Erythrocles schlegelii*) | 45.3 | 0.7 | <0.01 |
| Chinese perch (*Siniperca chuatsi*) | 20.7 | 1.5 | <0.01 |
| Scalpel sawtail  (*Prionurus scalprum*) | 22.1 | 1.4 | <0.01 |
| Red drum  (*Sciaenops ocellatus*) | 25.7 | 1.2 | <0.01 |
| Onespot snapper  (*Lutjanus monostigma*) | 15.4 | 1.9 | <0.01 |
| Silver sea meagre  (*Pennahia argentata*) | 15.3 | 1.8 | <0.01 |
| Croaker (*Larimichthys polyactis*) | 11.5 | 2.3 | <0.01 |
| White amur  (*Ctenopharyngodon idella*) | 3.1 | 8.3 | <0.01 |
| Sidespot goatfish  (*Parupeneus pleurostigma*) | 14.3 | 1.8 | <0.01 |
| Queenfish (*Scomberoides commersonnianus*) | 22.5 | 1.1 | <0.01 |
| Perch (*Lates calcarifer*) | 15.0 | 1.5 | <0.01 |
| Rock bullseyes  (*Priacanthus cruentatus*) | 35.4 | 0.6 | <0.01 |
| Scale carp  (*Cyprinus carpio carpio*) | 6.6 | 3.2 | <0.01 |
| Puntat (*Clarias fuscus*) | 1.5 | 13.8 | <0.01 |
| Tilefish (*Branchiostegus japonicus*) | 20.3 | 1.0 | <0.01 |
| Silver sillago (*Sillago sihama*) | 16.3 | 1.2 | <0.01 |
| Sailfin rubberlip  (*Diagramma pictum*) | 14.9 | 1.2 | <0.01 |
| Red snout emperor  (*Lethrinus lentjan*) | 19.8 | 0.9 | <0.01 |
| Popamno (*Trachinotus blochii*) | 21.6 | 0.8 | <0.01 |
| White flower croaker  (*Nibea albiflora*) | 23.9 | 0.7 | <0.01 |
| Bombay duck  (*Harpadon nehereus*) | 30.2 | 0.5 | <0.01 |
| Patterned tongue-sole (*Paraplagusia bilineata*) | 18.8 | 0.8 | <0.01 |
| Red tilefish  (*Branchiostegus japonicus*) | 24.8 | 0.6 | <0.01 |
| Common dolphinfish  (*Coryphaena hippurus*) | 29.8 | 0.5 | <0.01 |
| India snapper  (*Etelis carbunculus*) | 18.4 | 0.8 | <0.01 |
| Queensland grouper  (*Epinephelus lanceolatus*) | 1.7 | 8.2 | <0.01 |
| Nile tilapia  (*Oreochromis niloticus*) | 3.0 | 4.7 | <0.01 |
| Gurnard  (*Dactyloptena gilberti*) | 27.0 | 0.5 | <0.01 |
| Sliver grunt  (*Pomadasys argenteus*) | 10.8 | 1.2 | <0.01 |
| Rice field eel  *(Monopterus albus*) | 9.3 | 1.4 | <0.01 |
| Mi-juy croaker  (*Miichthys miiuy*) | 25.3 | 0.5 | <0.01 |
| Red mouthbeeder  (*Fistularia petimba*) | 3.6 | 3.3 | <0.01 |
| Monkfish (*Lophiomus setigerus*) | 23.5 | 0.5 | <0.01 |
| Rough flutemouth  (*Fistularia petimba*) | 23.5 | 0.5 | <0.01 |
| Needle fish (*Tylosurus crocodilus crocodilus*) | 38.4 | 0.3 | <0.01 |
| Rock grouper (*Epinephelus fasciatomaculosus*) | 21.8 | 0.5 | <0.01 |
| Sea barbel (*Arius maculatus*) | 20.8 | 0.5 | <0.01 |
| Overcast grouper  (*Cephalopholis boenak*) | 34.5 | 0.3 | <0.01 |
| Panther grouper  (*Cromileptes altivelis*) | 16.5 | 0.6 | <0.001 |
| Blue spotted rockod (*Cephalopholis miniata*) | 19.6 | 0.5 | <0.001 |
| Red snapper  (*Etelis carbunculus*) | 27.5 | 0.3 | <0.001 |
| Aulopus (*Hime japonica*) | 40.0 | 0.2 | <0.001 |
| Snakehead (*Channa asiatica*) | 1.5 | 4.5 | <0.001 |
| Sicklefish (*Drepane punctata*) | 22.6 | 0.3 | <0.001 |
| Rock fish  (*Sebastiscus marmoratus*) | 32.4 | 0.2 | <0.001 |
| Spotted half beak  (*Hemiramphus far*) | 21.6 | 0.3 | <0.001 |
| Scarbreast tuskfin  (*Choerodon azurio*) | 25.0 | 0.2 | <0.001 |
| Tuna (*Thunnus albacares*) | 42.1 | 0.1 | <0.001 |
| Sickle pomfret (*Taractichthys steindachneri*) | 41.0 | 0.1 | <0.001 |
| Lavender jobfish  (*Pristipomoides sieboldii*) | 40.0 | 0.1 | <0.001 |
| Pink snapper  (*Pristipomoides filamentosus*) | 40.0 | 0.1 | <0.001 |
| Stingray (*Hemitrygon akajei*) | 37.1 | 0.1 | <0.001 |
| Yellowing flyingfish  (*Cypselurus poecilopterus*) | 36.2 | 0.1 | <0.001 |
| Blowfish  (*Lagocephalus lunaris*) | 36.0 | 0.1 | <0.001 |
| Basa fish  (*Pangasianodon hypophthalmus*) | 2.0 | 1.7 | <0.001 |
| Luna lion fish (*Pterois lunulata*) | 33.0 | 0.1 | <0.001 |
| Squirrelfish  (*Neoniphon aurolineatus*) | 26.0 | 0.1 | <0.001 |
| Zander (*Sander lucioperca*) | 23.0 | 0.1 | <0.001 |
| Peacock grouper  (*Cephalopholis argus*) | 5.4 | 0.1 | <0.001 |
| Ocean sunfish (*Mola mola*) | 1.0 | 0.4 | <0.001 |
